# Study of Robustness in Functionally Identical Coupled Networks against Cascading Failures

**DOI:** 10.1371/journal.pone.0160545

**Published:** 2016-08-05

**Authors:** Xingyuan Wang, Jianye Cao, Xiaomeng Qin

**Affiliations:** Faculty of Electronic Information and Electrical Engineering, Dalian University of Technology, Dalian, 116024, China; Universidad Rey Juan Carlos, SPAIN

## Abstract

Based on coupled networks, taking node load, capacity and load redistribution between two networks into consideration, we propose functionally identical coupled networks, which consist of two networks connected by interlinks. Functionally identical coupled networks are derived from the power grid of the United States, which consists of many independent grids. Many power transmission lines are planned to interconnect those grids and, therefore, the study of the robustness of functionally identical coupled networks becomes important. In this paper, we find that functionally identical coupled networks are more robust than single networks under random attack. By studying the effect of the broadness and average degree of the degree distribution on the robustness of the network, we find that a broader degree distribution and a higher average degree increase the robustness of functionally identical coupled networks under random failure. And SF-SF (two coupled scale-free networks) is more robust than ER-ER (two coupled random networks) or SF-ER (coupled random network and scale-free network). This research is useful to construct robust functionally identical coupled networks such as two cooperative power grids.

## 1 Introduction

As a result of the constant progress of the society and the development of science and technology, complex phenomena in complex networks have received more attention [1–9] in the last decades. The Small-World model [4] (Watts and Strogatz, 1998) and the BA scale-free network model [5] (Barabási and Albert 1999) represented well-known landmarks and contributions in the last century. After that, the research about complex network is rising. One of the most important trends of research in the field is that of cascading failures. A cascade failure is a particular type of system failure, one or more damaged nodes (or edges) lead other ones to consequent failures as a result of coupling relationship between nodes (or edges), resulting in the collapse of the whole network. Considering the great harm of cascading failures, many researchers established models based on real networks to put forward preventive and controlled method.

The vast majority of researches on cascading failures have concentrated exclusively on isolated networks, ignoring the fact that many real-world networks interact with each other. An example of this tendency is represented by the framework of analysis of cascading failures in interdependent networks [10–15] proposed by Buldyrev et. al. [15].

They found threshold value *p*_*c*_ is subjected to degree distribution. In single network, broader degree distribution causes smaller *p*_*c*_. But in interdependent networks, broader degree distribution causes bigger *p*_*c*_, because high-degree nodes of one network can depend on low-degree nodes of the other. In a different perspective, Gao et al. [16] studied the dynamics of the cascades of failures in *n* coupled interdependent networks under a random initial attack on one of the networks. And then they found, for any tree-like network of networks, the critical percolation threshold and the mutual giant component depend only on the number of networks but not on the topology. In the same token, Parshani et al. [17] showed that reducing the coupling between interdependent networks leads to a change from the first order percolation phase transition to the second order percolation transition at a critical point. In consonance with these lines of research, Huang et al. [18] studied the robustness [19–24] of interdependent networks under tunable degree-targeted attacks. They found that interdependent networks are difficult to defend by strategies such as protecting the high degree nodes. In the same vein, Schneider et al. [25] proposed a systematic strategy for selecting a minimum number of autonomous nodes that reduce the chances of a catastrophic cascading failure. Wang et al. [26] introduced load, load redistribution, and node capacity to interdependent networks, and investigated the robustness of interdependent networks with different inter-linkages. They found that the different inter-linkages have a dramatic effect on the robustness of interdependent networks.

Their researches are based on coupled networks, in which two subnets are interdependent. But another kind of coupled networks is rarely studied, in which two subnets have the same function, and we call it functionally identical coupled networks. An example of functionally identical coupled networks is two interconnected grids. In America, the grid comprises many independent grids and many power transmission lines intended to interconnect them. Therefore, study on robustness of functionally identical coupled networks is important. Therefore, in this work, two power grids are considered as two independent networks and power transmission lines as the interlinks connecting them. We propose this configuration as functionally identical coupled networks and we aim at studying its robustness.

The interdependent networks and the functionally identical coupled networks are fundamentally different, by two main reasons.

(1) The functions of two sub-networks in interdependent networks are interdependent. But in functionally identical coupled networks, they are the same. (2) In interdependent networks, a failed node in one of them causes a failure in the corresponding node of the other network. But in functionally identical coupled networks, the only reason a node crashes is that it cannot handle the extra load from nodes directly connected to it.

The rest of this paper includes these sections: Cascading failures model is introduced in section 2. And in section 3, we show the experimental results and analysis of robustness. Finally, in section 4, we summarize our paper.

The aims of this paper are to study the effect of the broadness and average degree of the degree distribution on robustness in functionally identical coupled networks and single network under random attack. We compare the robustness between SF-SF, SF-ER and ER-ER, and then help to construct robust functionally identical coupled networks.

## 2 Cascading Failures Model of Functionally Identical Coupled Networks

For the sake of simplicity, for two networks *A* and *B* with the same size *N*, *A*_*i*_ (1≤*i*≤*N*) denotes a node in network *A*, and *B*_*i*_ (1≤*i*≤*N*) denotes a node in network *B*. The degree $k_{A_{i}}$ of node *A*_*i*_ is the number of nodes connected to *A*_*i*_ in network *A*; and the degree $k_{B_{i}}$ of node *B*_*i*_ is the number of nodes connected to *B*_*i*_ in network *B*. Every node in network *A* links only one node in network *B* and vice-versa. We assume that node *A*_*i*_ is linked to node *B*_*i*_ in network *B* and vice-versa.

In every network, each node has a load and a finite capacity, in the same way that in a power grid each power station has a load and a finite capacity. The node’s load corresponds to the intensity of electric current flowing in a power station, whereas its capacity corresponds to the biggest load that a power station can handle before breaking down. Generally, a higher-degree node carries a higher load. We assume that the initial load of a node is equal to its degree, and that its capacity is proportional to its initial load [23]. Consequently, initial load of node *A*_*i*_ in network *A* is defined as $L_{A_{\text{i}}}=k_{A_{i}}$ and initial load of node *B*_*i*_ in network *B* as $L_{B_{\text{i}}}=k_{B_{i}}$. Similarly, node’s *A*_*i*_ capacity in network *A* is defined as $C_{A_{i}}=(1+\beta )k_{A_{i}}$ and node’s *B*_*i*_ capacity in network *B* as $C_{B_{i}}=(1+\beta )k_{B_{i}}$, where *β* is the tolerance parameter, and *β*>0 [23].

The strategy that we propose here consists in randomly removing one node from functionally identical coupled network. For example, consider that a node *A*_*i*_ is removed. Then, its load will be redistributed among its neighboring nodes. However, the new extra-load on these neighboring nodes may exceed their capacity, causing them to fail. These new failed nodes provoke the new load redistribution. After several iterations, network is divided into many clusters. In the same way that in a power grid, if one power station is failed, electricity demand of the region will be supported from the surrounding power station connected to the failed one. Load redistribution may lead cascading failures.

We define the set of neighboring nodes of *A*_*i*_ as $\Gamma_{A_{i}}$, which consists of node *B*_*i*_ in network *B*, and neighboring nodes of *A*_*i*_ in network *A*. We assume that the extra load received by a node in $\Gamma_{A_{i}}$ from *A*_*i*_ is proportional to its degree. In consequence, the extra load $\Delta L_{A_{j}A_{i}}$ received by the neighboring node *A*_*j*_($A_{j}\in \Gamma_{A_{i}}$) in network *A* is:

$$\Delta L_{A_{j}A_{i}}=L_{A_{i}}k_{A_{j}}/\sum_{A_{a}\in \Gamma_{A_{i}}}k_{A_{a}},$$

and the extra load $\Delta L_{B_{i}A_{i}}$ received by neighboring node *B*_*i*_($B_{i}\in \Gamma_{A_{i}}$) in network *B*

$$\Delta L_{B_{i}A_{i}}=L_{A_{i}}k_{B_{i}}/\sum_{A_{a}\in \Gamma_{A_{i}}}k_{A_{a}}.$$

**Fig 1:**
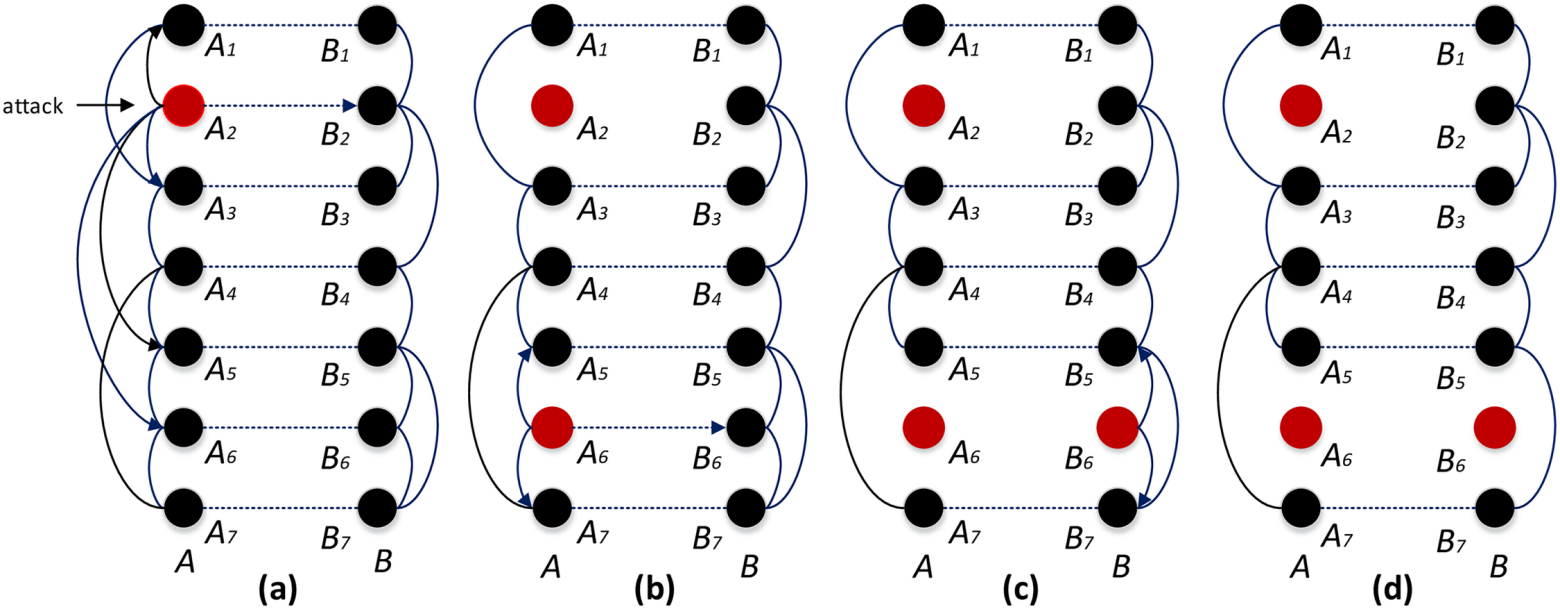
Iterative process of cascading failures in functionally identical coupled networks. As is shown above, two networks *A* and *B*, network *A* consists of 7 nodes, *A*_1_ −*A*_7_, and network *B* consists of 7 nodes, *B*_1_ − *B*_7_. Links between two networks are shown as horizontal straight dash lines, and A-links and B-links are shown as arcs. Arrowed arcs lines represent load redistribution from failed node to normal node. The failed nodes and the normal nodes are shown as solid red circle and solid black circle respectively. **1.** Disabling *A*_2_, and then load on *A*_2_ flow from it to *A*_1_, *A*_3_, *A*_5_, *A*_6_ and *B*_2_. **2.** Lines linked *A*_2_ are removed. Assume that load on node *A*_6_ exceeds its capacity after receiving the extra load from node *A*_2_, *A*_6_ fails and turns to red, which leads to load redistribution from *A*_6_ to *A*_5_, *A*_7_, and *B*_6_. **3.** Lines linked *A*_6_ are removed. Assume that *B*_6_ cannot handle the extra load from *A*_6_, *B*_6_ fails and turns to red. Load on *B*_6_ is redistributed to *B*_5_ and *B*_7_. **4.** No further node is overloaded, and iterative process is over.

The process of cascading failures is illustrated in Fig 1. Assuming that the cascading failures are induced by removing node *i*, and that the size of normal nodes in functionally identical coupled networks is *S*_*i*_ after a cascade failure, we use *S* = ∑_*i*∈*A*∪*B*_*s*_*i*_/(2*N*)^2^ to characterize the robustness of functionally identical coupled networks.

## 3 The Analysis of the Robustness

We construct three types of functionally identical coupled networks: the ER-ER network (Erdös-Rényi random network [6–7]), the SF-ER network and the SF-SF (SF is BA scale-free network [5], which is generated by an algorithm proposed by Dorogovtsev [27]).

Our model is implemented by Java programming language running on a PC with a 3.1 GHz processor, 6.0 GB memory and Windows 7 operating system. Data of the western United States power grid (S1 File) used in Part 3.3 was compiled by Duncan Watts and Steven Strogatz [4].

### 3.1 Comparison of the robustness between the functional consistency coupling network and single network

**Fig 2:**
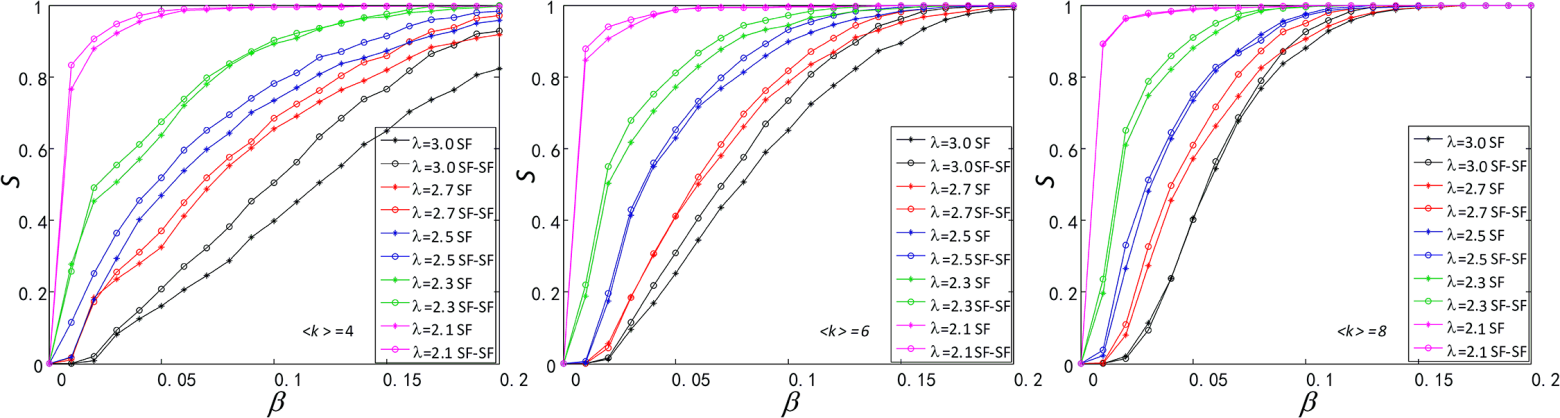
Comparison of robustness between functionally identical coupled networks (SF-SF) and single network (SF). In SF-SF and SF, the three SF networks have the same size *N* = 1000, the same average degree 〈*k*〉_*SF*_ ∈ {4, 6, 8}, the same degree distribution exponent *λ* ∈ {2.1, 2.3, 2.5,2.7, 2.7, 3.0}.

**Fig 3:**
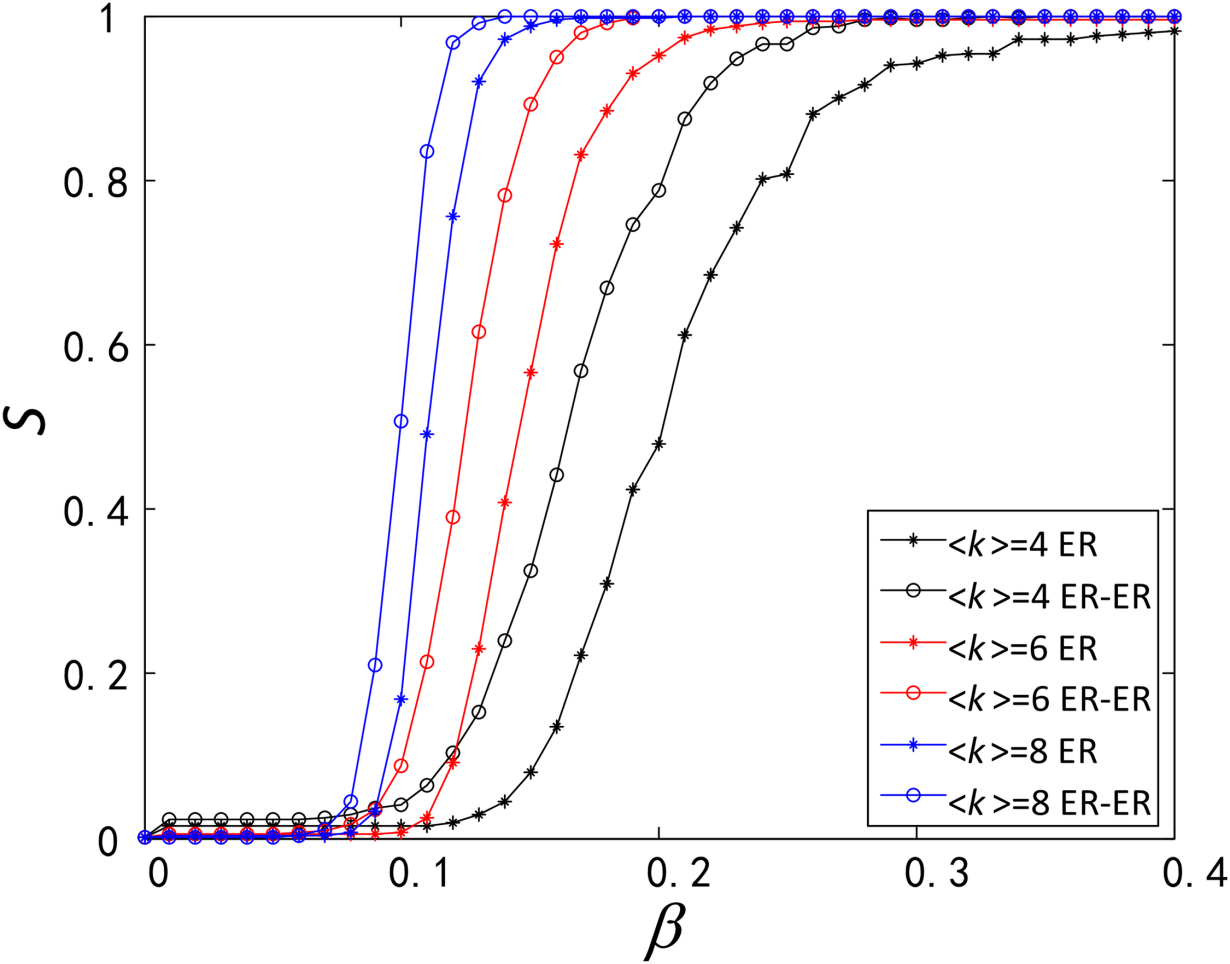
Comparison of robustness between functionally identical coupled networks (ER-ER) and single network (ER). In ER-ER and ER, three ER networks have the same size *N* = 1000, the same average degree 〈*k*〉_*ER*_ ∈ {4, 6, 8}.

In this section, we address to study the robustness of SF-SF and ER-ER against cascading failures in Figs 2 and 3. We also apply the same cascading failure process to two types of single network SF and ER, and compare the robustness of SF-SF and SF as well as of ER-ER and ER.

In two types of single network SF and ER, the load of a node is set as double of its degree, and then the functionally identical coupled networks and the single network have the same total capacity and load. Based on the same total load and capacity, we compare the difference of robustness between them as showed in Figs 2 and 3.

As is shown in Fig 2, we compare the size of normal nodes *S* between the SF-SF and SF after cascading failures. We find that the SF-SF network has stronger robustness than the SF network. Therefore, dividing a single SF or ER network into a SF-SF or ER-ER networks is useful for better robustness, and among the possible reasons are:

1. Their average degree is different. Three SF networks in the SF-SF and SF have the same average degree and size, and there are one-to-one interlinks between two SF networks in the SF-SF. And it appears clear that 〈*k*〉_*SF − SF*_ = 〈*k*〉_*SF*_ + 1 (〈*k*〉_*SF − SF*_ is average degree of the SF-SF). Therefore, if a node in the SF-SF is overloaded, the neighbor nodes will receive lesser extra-load from it. And the lesser the load, the lower the overload probability.
2. Interlinks between two subnets can make full use of the network capacity. If a node is overloaded and load on it can be distributed to the other nodes in the whole network, the probability of occurrence of cascading failures will be greatly reduced. So, if one node in the SF-SF is overloaded, its load can be reassigned to other nodes connected to it by interlink, making full use of the capacity of the other node’s capacity.

We also find that when the degree distribution exponent *λ* decreases, the robustness of both the SF-SF and SF increases, due to that the number of nodes with small degree increases with the decrease of *λ*. The failure of nodes with small degree does not has a big impact on the network.

Like in the comparison of robustness between the SF-SF and SF, we also find that the ER-ER network has a stronger robustness than the ER network (See Fig 3), due to the same reasons discussed for the SF-SF and SF networks. Lastly, as we can see in Figs 2 and 3, robustness has been enhanced in the SF-SF, SF, ER-ER and ER networks with the increase of 〈*k*〉_*SF*_ and 〈*k*〉_*ER*_.

In fact, in our model, robustness of single SF or ER network is invariable as long as the initial load of nodes is proportional to their degree. Therefore, in a single SF or ER, the initial load of nodes is equal to their degree or double degree, which does not affect their robustness. In consequence, merging two same single SF or ER networks into SF-SF or ER-ER is also useful for better robustness.

### 3.2 The comparison of the robustness between SF-SF, SF-ER and ER-ER

In this part, we compare the robustness of three types of functionally identical coupled networks SF-SF, SF-ER and ER-ER against cascading failures.

**Fig 4:**
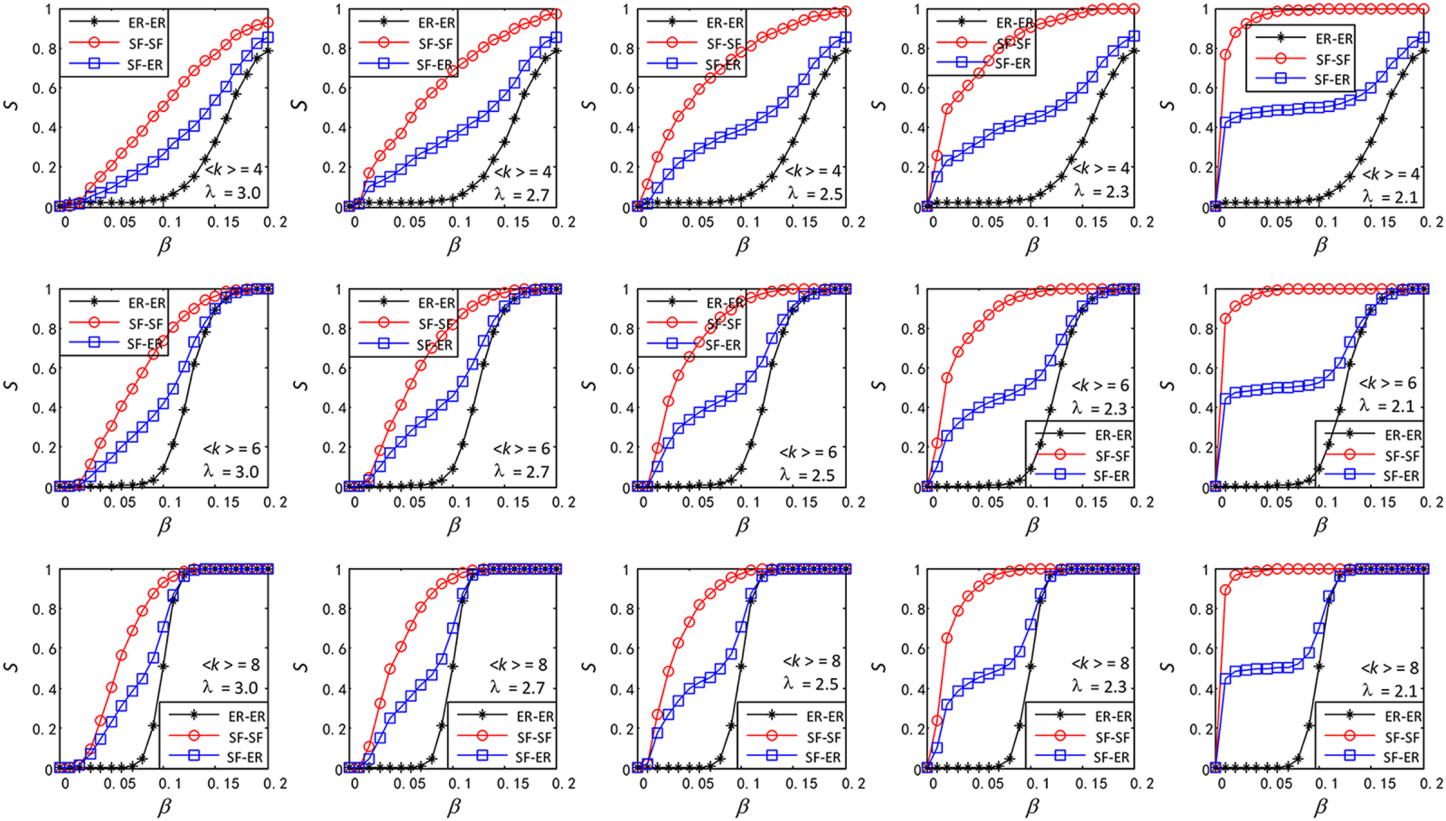
Comparison of robustness between three types of functionally identical coupled networks (SF-SF, SF-ER and ER-ER). Every subnet in SF-SF, SF-ER and ER-ER has the same average degree, 〈*k*〉_*ER*_ = 〈*k*〉_*SF*_ = 〈*k*〉 ∈ {2, 4, 8}, same size *N* = 1000, and every SF in SF-SF, SF-ER has the same degree distribution exponent *λ* ∈ {2.1, 2.3, 2.5, 2.7, 3.0}.

**Fig 5:**
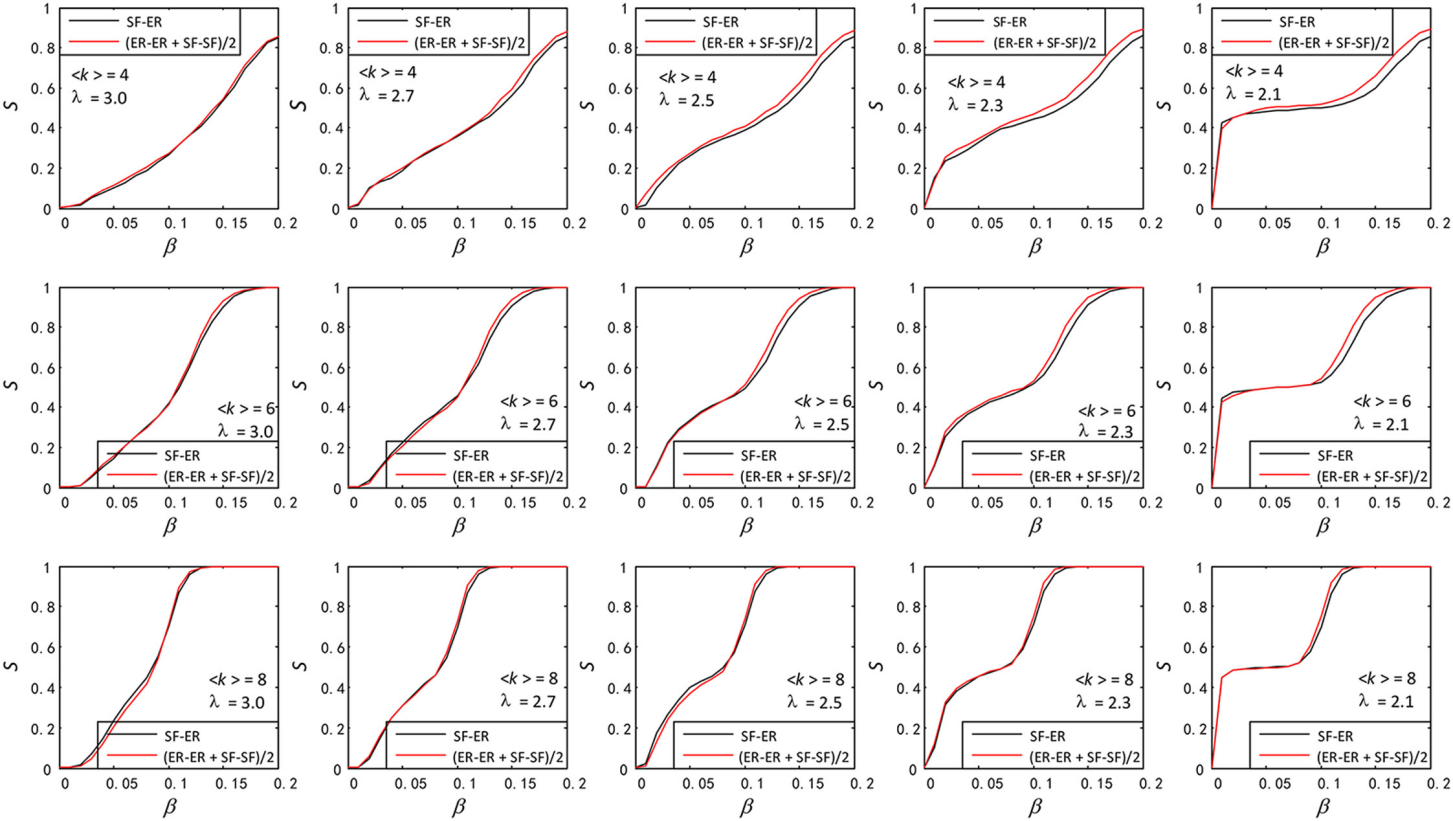
Comparison of the *S* between SF-ER and the average of SF-SF and ER-ER.

We find, as shown in Fig 4, that the order of robustness for a different average degree of 〈*k*〉 = 2, 4, 8 and a degree distribution exponent *λ* = 2.1, 2.3, 2.5, 2.7, 3.0 is SF-SF>SF-ER>ER-ER, as shown in the last column of Fig 4. We also find that with the growth of *β*, *S*_*SF* − *ER*_ grows only when *S*_*SF* − *SF*_ or *S*_*ER* − *ER*_ grows. *S*_*SF* − *ER*_, *S*_*SF* − *SF*_ and *S*_*ER* − *ER*_ represent the size of normal nodes in SF-ER, SF-SF, ER-ER after cascading failures. Therefore, we compare *S*_*SF* − *ER*_ and the average of *S*_*SF* − *SF*_ and *S*_*ER* − *ER*_ in Fig 5. Amazingly, we find that *S*_*SF* − *ER*_ and the average of *S*_*SF* − *SF*_ and *S*_*ER* − *ER*_ are closed.

### 3.3 Application of the model in power grid

**Fig 6:**
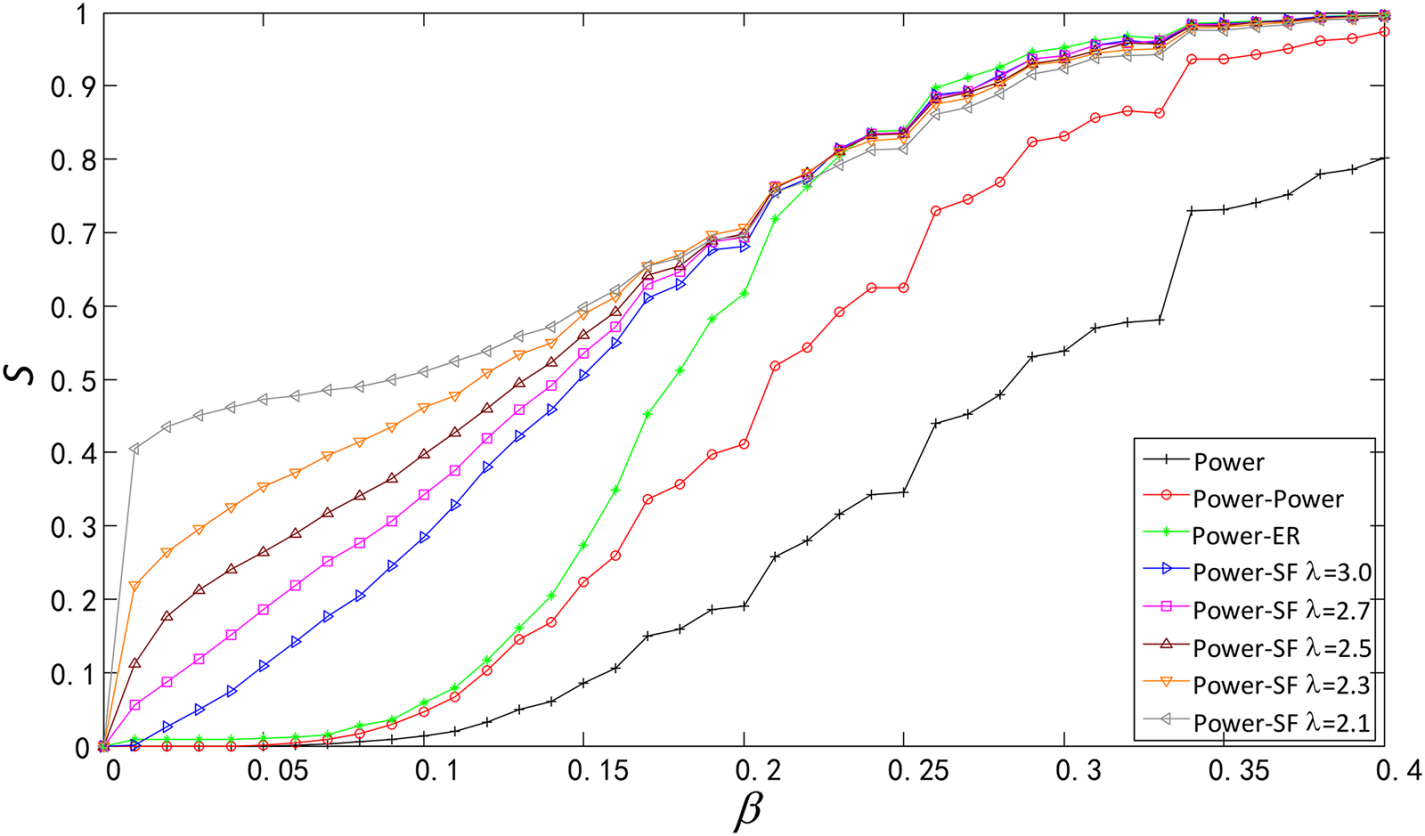
Comparison of robustness among Power, Power-Power, Power-ER and Power-SF. The Power network in this figure is the western United States power grid, which is an undirected network with 4941 nodes and 6594 edges. Except the Power network, ER and SF network contain 4941 nodes, the average degree of them is 4 and the degree distribution exponents of SF are *λ* ∈ {2.1, 2.3, 2.5, 2.7, 3.0}.

Secondly, we apply our model to a infrastructure network, the western United States power grid, and compare the robustness among the Power-Power, a single Power, Power-ER and Power-SF (see Fig 6).

Similarly, in single network we set the load of a node as double of its degree, and then functionally identical coupled networks and single network have the same total capacity and load.

As shown in Fig 6. The Power-Power is more robust than the single Power against cascading failures. This is the same conclusion drawn in section 3. With the exception of the Power-Power and the single Power, the order of robustness is: Power-SF (*λ* = 2.1) > Power-SF (*λ* = 2.3) > Power-SF (*λ* = 2.5) > Power-SF (*λ* = 2.7) > Power-SF (*λ* = 3.0) > Power-ER, broader degree distribution, stronger robustness.

## 4 Conclusions

Many researchers study the robustness based on interdependent networks, in which each node depends on another. But few people consider the function consistency of coupled networks. In this paper, we fill this gap and study the robustness of functionally identical coupled networks against cascading failures under random attack. In section 3.1, we find that the SF-SF or ER-ER is more robust than SF or ER under random attack. In simple terms, in order to improve the robustness of two single SF or ER networks under random attack, we should couple them to SF-SF or ER-ER networks. We also can divide a single SF or ER network into a SF-SF or ER-ER networks for better robustness. Then, increase of *λ* and the average degree enhances the robustness in SF-SF and SF networks. In section 3.2, Fig 4 tells us that we should couple two single networks as SF-SF for stronger robustness instead of SF-ER or ER-ER. Finally, as is shown in Fig 5, the *S*_*SF* − *ER*_ and the average of the *S*_*SF* − *SF*_ and *S*_*ER* − *ER*_ are closed. We hope these findings will be useful to construct robust functionally identical coupled networks.

## Supporting Information

S1 File Western States Power Grid. (NET)
